# Tourism may trigger physiologically stress response of a long-term habituated population of golden snub-nosed monkeys

**DOI:** 10.1093/cz/zoaa076

**Published:** 2020-12-15

**Authors:** Haochun Chen, Hui Yao, Xiangdong Ruan, Bernard Wallner, Julia Ostner, Zuofu Xiang

**Affiliations:** 1 College of Life Science and Technology, Central South University of Forestry & Technology, Changsha, 410004, China; 2 Shennongjia National Park, Hubei Province, Shennongjia Forest District, Hubei, 442411, China; 3 Academy of Forest Inventory and Planning, State Forestry and Grassland Administration, Beijing, 100714, China; 4 Department of Behavioural Biology, Faculty of Life Sciences, University of Vienna, Vienna, 1090, Austria; 5 Department for Behavioral Ecology, University of Göttingen, Kellnerweg 6, Göttingen, D-37077, Germany

Wildlife tourism is a thriving form of nature-based tourism that can provide revenue for conservation funds, and increase public awareness of biodiversity conservation (Xiang et al. 2011). Tourism, however, may induce behavioral and physiological stress responses on animals (Maréchal 2015; Beehner and Bergman 2017). Part of the physiological stress response is increased activity of the hypothalamic–pituitary–adrenal (HPA) axis, resulting in the systemic elevation of glucocorticoids. An increase of glucocorticoids in the bloodstream leads to rapid mobilization of stored energy reserves and inhibitions of nonimmediately critical activities like growth, reproduction, and digestion in response to environmental challenges (Beehner and Bergman 2017). Although there is a debate as to whether deleterious chronic stress exists in wild populations (Beehner and Bergman 2017), measuring glucocorticoids is an important tool in assessing anthropogenic disturbance to animals (Maréchal 2015; Palme 2019). Cortisol, the main glucocorticoid secreted in primates, is metabolized by the liver and partly excreted in urine, meaning that elevations of cortisol and its metabolites produced in the stress response can be detected by using urine (Palme 2019).

The golden snub-nosed monkey *Rhinopithecus roxellana* is 1 of 5 snub-nosed monkey species belong to the subfamily Colobinae (Xiang 2020). Snub-nosed monkeys are very photogenic and captivating animals that have recently received increased attention in the media, such as featuring in the Disney documentary, “Born in China” (Lu 2017). There are at least 5 sites in China where snub-nosed monkey tourism programs either exist or are likely to be launched in the near future. They have the capacity to be a major tourist attraction, which could draw a large number of visitors from all over the world. Every snub-nosed monkey tourist area would like to market itself as “eco-tourism” or “sustainable tourism.” A group of golden snub-nosed monkeys at Dalongtan, Shennongjia National Park have been visited by tourists since 2007 (Xiang et al. 2011). These habituated monkeys are frequently visited by dozens to hundreds of tourists. We hypothesize that these monkeys are physiologically stressed from tourism, and we test this by examining potential correlations between urinary cortisol concentration (CC) and the intensity of tourism activity. We measure tourism activity from different aspects: 1) number of tourists—the total number of tourists that visited this group of monkeys over a given day; 2) exposure time to tourists (%)—the percentage of the scans in which tourists were present at the viewing area during the visiting hours on a given day; 3) tourist distance (m)—the mean distance estimated in meters between tourists and target individuals in all scans in which tourists were present on a given day. Details of methods are described in Supplementary Materials.

We collected 272 urine samples from 16 individuals (3 males and 13 females) in 49 days when at least 1 tourist was present. The means of urinary CC of males and females were 205.348 ± 129.286 ng/mg creatinine (CR, *n = *35), and 213.473 ± 133.304 ng/mg CR (*n = *237), respectively. The corresponding measures of intensity of tourism activity were as follows. Average number of tourists was 65.0 ± 58.0 (mean ± SD, *n = *49). Average exposure time to tourist was 39.6 ± 21.0% (*n = *49). Average tourist distance was 20.4 ± 10.0 m (*n = *272). We fitted linear mixed models to explain the variations in urinary CC. The full model included the time when samples were voided, sex of the monkey, tourist-related variables (number of tourists, exposure time to tourists, and tourist distance), and interactions between sex and tourist-related variables as fixed factors. IDs of individuals were entered into the model as a random factor. We used the stepwise model selection method. According to the best model, exposure time to tourists and tourist distance were the factors that explain the variation in the urinary CC (Table 1). Urinary CC was positively correlated to exposure time to tourists (*t *=* *11.57, *P < *0.001), and negatively correlated to tourist distance (*t* = −5.84, *P < *0.001).

**Table 1.** Details of the best model considered as explanatory for urinary cortisol variation in golden snub-nosed monkeys (95% confidence intervals in brackets)

Term	Estimate	SE	*t*	*P*
Intercept	5.179 (5.040, 5.319)	0.069	74.826	<0.001
Exposure time	0.304 (0.253, 0.356)	0.026	11.568	<0.001
Tourist distance	−0.154 (−0.205, −0.102)	0.026	−5.842	<0.001
Random	Variance	SD		
ID	0.066	0.257		

Our results confirmed the prediction that tourism could lead to elevation of cortisol secretion in habituated golden snub-nosed monkeys. Contrary to our expectation, urinary CC was not associated with the number of tourists. Urinary CC of the monkeys increased as exposure time to tourists increased (Figure 1A). With decreasing tourist distance, there was a significant increase in the measured urinary CC (Figure 1B). Tourists occasionally violated park regulations sometimes, getting too close to the monkeys, even touching them. These monkeys may perceive tourists in close proximity as a threat. A similar result was found in habituated gorillas *Gorilla gorilla gorilla* (Shutt et al. 2014): fecal glucocorticoid metabolites were positively correlated to the frequency that tourists violated 7 m distance regulation. Tourist visiting time and distance to the golden snub-nosed monkeys need to be limited to minimize the potentially detrimental effects of tourism. This calls for exercising caution when nonhuman primate tourism projects are undertaken at other sites.

**Figure 1.**
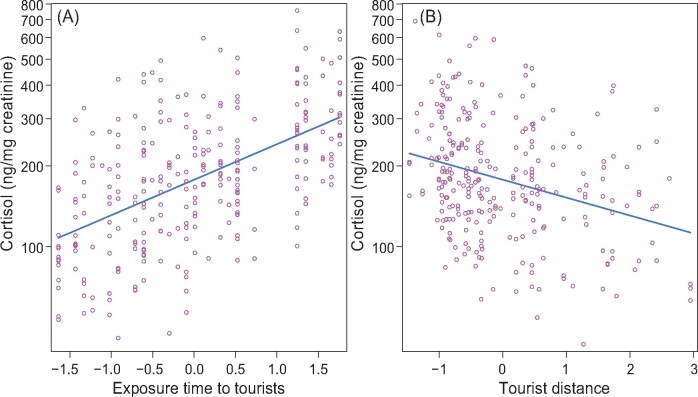
Effect plots showing linear mixed model-predicted changes in urinary CC with respect to exposure time to tourists (**A**, *z*-transformed) and tourist distance (**B**, *z*-transformed). Open circles correspond to partial residuals.

## Funding

Financial support was provided by the National Natural Science Foundation of China (31870509 and 31670397).

## Supplementary Material

zoaa076_Supplementary_Data
