# Supplementary material for: Tourism may trigger physiologically stress response of a long-term habituated population of golden snub-nosed monkeys
Source: Curr Zool. 2020 Dec 15;67(4):465–7. doi: 10.1093/cz/zoaa076 (PMC8489030; doi:10.1093/cz/zoaa076)
Supplement: zoaa076_Supplementary_Data [file zoaa076_supplementary_data.zip › Supplementary_Materials.docx]

Tourism may trigger physiologically stress response of a long-term habituated population of golden snub-nosed monkeys

Haochun CHen^a^, Hui Yao^b^, Xiangdong Ruan^c^, Bernard Wallner^d^, Zuofu Xiang^a, c*^

^a^College of Life Science and Technology, Central South University of Forestry & Technology, Changsha 410004, China; ^b^Shennongjia National Park, Hubei Province, Shennongjia Forest District, Hubei 442411, China; ^c^Academy of Forest Inventory and Planning, State Forestry and Grassland Administration, Beijing 100714, China; ^d^Department of Behavioural Biology, Faculty of Life Sciences, University of Vienna, 1090 Vienna, Austria

*Address correspondence to Zuofu Xiang. E-mail: xiangzf@csuft.edu.cn

Handling editor:  Zhi-Yun JIA

Received on 3 November 2020; accepted on 8 December 2020

Key Words: Tourism, *Rhinopithecus roxellana*, cortisol, habituated, stress

# Supplementary Materials

## Materials and Methods

## Study site and study subject

We conducted the study at Dalongtan (31°29΄N, 110°18΄E; 2,200 m *asl*) in Shennongjia National Park, central China. This area is a highly seasonal temperate forest (Xiang et al., 2019; Zhang et al., 2019).

In January 2006, the local staff had habituated and began provisioning about 30 wild golden snub-nosed monkeys, including several one-male, multi-female units (OMUs) and an all-male unit (AMU). Tourism then started in the following year. At Dalongtan the monkeys naturally eat leaves, bark, herbs, etc. (Li, 2006), and the complementary foods provided to them three times a day include apples, peaches, sweet potatoes, and peanuts. Their activity range is within a 400 - 500 m radius from the provisioning site. Several provisioning sites are utilized in rotation so that the vegetation consumed and destroyed by the monkeys has time to recover.

Tourists who visit Dalongtan to watch the monkeys must get out of their cars at least 400 m away from the provisioning site. They are required to wear provided camouflage clothing which has been UV disinfected. Between 2007 and 2014, tourists were provided with peanuts, one of the monkeys’ favorite foods, so they could encourage the monkeys to come closer. Usually, the males from the AMU took peanuts from the tourists’ hands. Individuals in OMUs stayed away from tourists on most occasions. Feeding by tourists was forbidden in 2015 because of injuries caused to humans by monkeys trying to get peanuts and due to the risk of pathogen and parasite transmission (Zhang et al*.*, 2017). Our study was carried out after the ban on tourist provisioning. To date, the local staff continues to offer individuals from both AMU and OMU peanuts to attract them closer to tourists if they are not near the viewing site or inactive on the trees. The visiting time is from 8:00 - 11:30 and 14:00 - 17:30, and most tourists visit during holiday periods, particularly during the summer holidays (June-August) when hundreds of tourists visited this area per day.

The AMU had left the tourist site before we began this study, and we were unable to collect samples from all the five remaining OMUs, so we chose three OMUs as focal units. Urine samples were collected from three resident males and 13 adult females in the selected OMUs. The individuals were identified based on facial features, body features (e.g. injures), and pelage color (Yu et al*.*, 2013).

## Tourist disturbance

Data were collected from 7 August to 23 October 2015. We counted every tourist present at the viewing area, and conducted scans every five minutes and the estimated distances between target individuals and tourists between 8:30 and 11:30 a.m. Distance estimates were recorded as 0-2 m, 2-5 m, 5-10 m, 10-15 m, 15-20 m, 20-30 m, 30-40 m, or 40-50 m, and the upper end of each range was used for further analyses. We adopted three different measures to quantify intensity of tourism activity: (a) number of tourists– the total number of tourists that visited this group of monkeys over a given day; (b) exposure time to tourists (%) – the percentage of the scans in which tourists were present at the viewing area during the morning visiting hours on a given day; (c) tourist distance (m) – the mean distance between tourists and target individuals in all scans in which tourists were present on a given day.

## Sample collection and analyses

According to our previous research (Chen et al*.*, 2017), it takes 3.5 h (SD = 1.6 h) between exposure to a stressor and the excretion of urinary cortisol excretes in captive golden snub-nosed monkeys. However, these provisioned monkeys urinate less frequently than captive ones, and, in particular, they seldom urinate at noon (12:00 – 13:30) based on our observation. Hence, urine samples collected between 13:30 and 16:30 were used for analyzing urinary cortisol concentration (CC). The time of the day when the sample was voided was included as a random effect to control for any potential diurnal pattern.

We waited beneath branches on which target monkeys were resting on the tree. Once a monkey urinated, we collected as much urine as possible using a bucket lined with a disposable plastic bag. The urine was then syringed into tubes that were labeled with the date, time, and the individual’s ID. The plastic bag was replaced with a new one between different samples. Samples were temporally being placed in a cool box with ice bags until they could be stored in the freezer (­− 20℃­) within four hours of collection. We discarded urine samples contaminated by feces or excreta of other monkeys. Samples were kept frozen until laboratory analysis that performed at Central South University of Forestry & Technology.

After thawing, urine samples were centrifuged at 4000 rpm for 15 min. The supernatant was then diluted, and the hormone concentration was assessed using a commercial enzyme immunoassay kit (EIA, catalog #K003-H5) from Arbor Assays (Ann Arbor, USA) which was validated in a previous study (Chen et al*.*, 2017). To adjust for variations in the water content of urine samples, we measured the urinary creatinine (Cr) using detection kits also from Arbor Assays (catalog #K002-H5). EIA results divided by creatinine values were then used to obtain standardized urinary CC. The sensitivity of cortisol and creatinine assays were 17.3 pg/mL and 0.019 mg/dL, respectively. Intra-assay and inter-assay coefficients were 7.1% (*n =* 5) and 9.3% (*n =* 5) respectively for cortisol assays, and 4.6% (*n* = 5) and 5.5% (*n =* 5) respectively for creatinine assays.

## Data analysis

Before applying any statistical models, data exploration was carried out following the protocol described by Zuur et al. (2010). Scatterplots between each continuous predictor variable and response variable were made to detect the type of relationships. Boxplots were used to visualize associations between response variable and categorical predictor variables. We fitted linear mixed models (LMM) using the ‘lmer’ function in ‘lmertest’ package (Kuznetsova et al*.*, 2017). The full models included the time when samples were voided, sex of the monkey, tourist-related variables (number of tourists, exposure time to tourists, and tourist distance), and interactions between sex and tourist-related variables as fixed factors. IDs of individuals were entered into the model as a random factors. All quantified independent variables were z-transformed by ‘scale’ function, and urinary CCs were transformed with the natural logarithm to meet the assumption of residuals normality. We calculated variance inflation factors using ‘vif’ function’ in ‘car’ package to identify collinear variables (VIF < 5 indicating acceptable collinearity). To identify the parsimonious model, we eliminated fixed factors one at a time, and used likelihood ratio chi-square tests to compare full and reduced models. A significant result indicated that the factor should not be eliminated. (Zuur et al*.*, 2009; Goundie et al*.*, 2015). For likelihood ratio test, models were fitted using maximum likelihood estimation, whereas parameter estimates and *p*-values were calculated using restricted maximum likelihood (Zuur et al*.*, 2009; Hunninck et al*.*, 2017). Model validations were visually inspected via residual plots following method Zuur et al. (2009) described. Analyses were conducted using R version 3.6.3, and statistical significance levels were set at *α* = 0.05. All means are reported with SDs.

# Animal Ethics

Prior to conducting this study, approval was obtained from the Shennongjia National Nature Reserve (snnr-081201), and the Institutional Animal Care and Use Committee of Central South University of Forestry & Technology (csuft-090120).

# References

Chen H, Yao H, Yang W, Fan P, Xiang Z, 2017. Assessing the utility of urinary and fecal cortisol as an indicator of stress in golden snub-nosed monkeys *Rhinopithecus roxellana*. *PeerJ* **5**: e3648.

Goundie ET, Rosen DAS, Trites AW, 2015. Dive behaviour can predict metabolic expenditure in Steller sea lions. *Conservation Physiology* **3**: cov052.

Hunninck L, Ringstad IH, Jackson CR, May R, Fossøy F, Uiseb K, Killian W, Palme R, Røskaft E, 2017. Being stressed outside the park—conservation of African elephants *Loxodonta africana* in Namibia. *Conservation Physiology* **5**: cox067.

Kuznetsova A, Brockhoff PB, Christensen RHB, 2017. lmerTest Package: Tests in Linear Mixed Effects Models. *Journal of Statistical Software* **82**: 1–26.

Li YM, 2006. Seasonal variation of diet and food availability in a group of Sichuan snub-nosed monkeys in Shennongjia Nature Reserve, China. *American Journal of Primatology* **68**: 217–233.

Xiang Z, Fan P, Chen H, Liu R, Zhang B, Yang W, Yao H, Grueter CC, Garber PA, Li M, 2019. Routine allomaternal nursing in a free-ranging Old World monkey. *Science Advances* **5**: eaav0499.

Yu Y, Xiang Z-F, Yao H, Grueter CC, Li M, 2013. Female snub-nosed monkeys exchange grooming for sex and infant handling. *PLoS ONE* **8**: e74822.

Zhang B, Ye P, Yao H, Yang W, Qin J, Xiang Z, 2017. The influence of ecotourism on the gastrointestinal parasites of golden snub-nosed monkeys *Rhinopithecus roxellana* (in Chinese). *Acta Theriologica Sinica* **37**: 374–382.

Zhang YJ, Chen YX, Chen HC, Chen Y, Yao H, Yang WJ, Ruan XD, Xiang ZF, 2019. Social functions of relaxed open-mouth display in golden snub-nosed monkeys *Rhinopithecus roxellana*. *Zoological Research* **40**: 113–120.

Zuur AF, Ieno EN, Elphick CS, 2010. A protocol for data exploration to avoid common statistical problems: Data exploration. *Methods in Ecology and Evolution* **1**: 3–14.

Zuur AF, Ieno EN, Walker NJ, Saveliev AA, Smith GM, 2009. *Mixed Effects Models and Extensions in Ecology with R*. Springer-Verlag, New York.
